# Evaluation of the Psychometric Properties of the 18-Items Dysfunctional Attitudes Scale (Form B) in a Portuguese Sample of People Aged 60 and Over

**DOI:** 10.3390/nursrep14030165

**Published:** 2024-09-03

**Authors:** Celso Silva, Rogério Ferreira, Bruno Morgado, Brooke C. Schneider, Ana João, Francisco Sampaio, Lara G. Pinho, César Fonseca

**Affiliations:** 1 Higher School of Health, Polytechnic Institute of Beja, 7800-295 Beja, Portugal; 2 Instituto de Investigação e Formação Avançada, University of Évora, 7000-811 Évora, Portugal; 3 Comprehensive Health Research Centre (CHRC), University of Évora, 7000-811 Évora, Portugal; 4 Escola de Doctorat, Universitat Rovira y Virgili, 43005 Tarragona, Spain; 5 Clinic for Psychiatry and Psychotherapy, University Medical Center Hamburg-Eppendorf, 20246 Hamburg, Germany; 6 Nursing Department, University of Évora, 7000-811 Évora, Portugal; 7 Nursing School of Porto, 4200-072 Porto, Portugal; 8 CINTESIS@RISE, Nursing School of Porto (ESEP), 4200-072 Porto, Portugal

**Keywords:** dysfunctional attitudes scale, 18 items (Form B), psychometric properties, validation, Portuguese

## Abstract

The aim of the study is to assess the psychometric properties of the Portuguese version of the 18-items Dysfunctional Attitudes Scale (Form B) in a Portuguese sample of people aged 60 and over. Background: The 18-item Dysfunctional Attitudes Scale (Form B) is an instrument for assessing dysfunctional attitudes and can be useful as a predictor of depression for both initial episodes and relapses Methods: This was a one-stage cross-sectional survey of Portuguese-speaking people aged 60 years and over, able to read and write, functionally autonomous, and living in the community in their usual residences. The instrument was evaluated for its psychometric properties. Convergent validity with the Beck Depression Inventory-II was assessed. Results: The Portuguese version of the 18-item Dysfunctional Attitudes Scale (Form B) was structured into three factors, which explained a total of 58.95% of the total variance of the instrument. The exploratory factor analysis resulted in a modified model in which three factors were obtained with an eigenvalue greater than one (Kaiser’s criterion). Three factors were obtained instead of two as in the original study. It showed an internal consistency (Cronbach’s alpha = 0.770) and an interclass correlation coefficient ranging from 0.166 to 0.449, and the overall convergent validity with the Beck Depression Inventory-II was considered good; Conclusions: The Portuguese version of the Dysfunctional Attitudes Scale (DAS-18B) of people aged 60 and over demonstrated good psychometric properties.

## 1. Introduction

Depression is considered a common mental disorder by the World Health Organisation [1]. The COVID-19 pandemic has increased depressive symptoms in older adults in general, as before the pandemic it was estimated that 5.7 per cent of older adults had depressive symptoms [2], and this percentage increased to 28.1% due to the COVID-19 pandemic [3]. In Portugal, the pandemic was responsible for 52.9 per cent of older adults having depressive symptoms [4]. A recent systematic literature review reports several reasons for the increase in depressive symptomatology in older adults during the COVID-19 pandemic, including feelings of vulnerability to the risk of contracting the disease, feelings about the high mortality caused by the pandemic, receiving news about COVID-19 from the media, having infected acquaintances, and containment measures to control the disease that led to increased social isolation [5].

In fact, the COVID-19 pandemic has significantly aggravated social isolation, and the social distancing measures implemented have led to increased feelings of loneliness and anxiety in this demographic group, which has caused, in many cases, a decline in mental well-being due to decreased social interactions and support networks [6], with one study highlighting that older adults reported high levels of depression and anxiety and that these reports were associated with the duration of their isolation during the pandemic [7].

Several studies attribute a central role to cognitions in the pathogenesis of depression, in which people use rules, schemas and assumptions to interpret situations relating to themselves, others, and the world. When these rules, schemes and assumptions are organised and structured in an inflexible, rigid, uncompromising, and often unrealistic way, they can lead to difficulties in adapting, which, in turn, leads to personal suffering. These negative cognitions or thinking styles are called dysfunctional attitudes [8,9,10,11,12].

The cognitive model of depression proposed by Beck [13] has two structuring elements, namely the cognitive triad and cognitive biases. The cognitive triad consists of (1) a negative view of oneself, in which the person sees themselves as inadequate or maladjusted; (2) a negative view of the world (e.g., others, relationships, work and activities); and (3) a negative view of the future. This cognitive triad makes the depressed person feel helpless and hopeless and promotes negative feelings in general (i.e., pessimism and depression). Cognitive biases represent systematic and recurrent errors in the perception, interpretation and processing of information, which result in hasty and erroneous conclusions regarding personal performance and the evaluation of everyday life situations (i.e., attentional preferences for negative information [13,14,15,16,17,18]). It was hypothesised that there is a reciprocal relationship between the cognitive triad and information-processing biases (e.g., preferential attention for negative information about oneself, such as perceived rejection by others, leads to increased feelings of worthlessness, as well as increased monitoring of future situations for rejection).

Depression stems from an association between negative thinking styles exemplified by the cognitive triad and stressful events [19]. These negative thinking styles have been termed dysfunctional attitudes, which are inflexible and maladjusted beliefs about the self, the world around the individual, the individual, and the future [20,21]. Depression, according to Beck’s model, occurs when a stressful life event activates negative schemas and cognitions, leading to dysfunctional attitudes [21]. Several studies have reported that more dysfunctional attitudes are associated with higher levels of depressive symptoms and, to the same extent, fewer dysfunctional attitudes are associated with lower levels of depressive symptoms [22,23,24,25].

Instruments to assess dysfunctional beliefs are relevant for research in psychotherapy and depression intervention, particularly for cognitive–behavioural approaches, as well as in clinical settings. A greater availability of such instruments could help improve the personalisation of interventions so that healthcare professionals can better target dysfunctional beliefs and depressive content that is personally relevant to patients. Given the increase in the prevalence of depression due to the COVID-19 pandemic [26], so much so that it rose from fifth to second place in terms of the causes of the decrease in the number of years lived with disability [3], speeding up and improving the effectiveness of depression treatment is important to improve the use of the limited mental health resources available. In addition, depression can be conceptualised as a chronic condition, as individuals with a history of depression have a high risk of relapse, and this risk increases with each subsequent episode [27,28,29]. Thus, a proper assessment of dysfunctional attitudes is important, since dysfunctional attitudes can be predictors of the onset and relapse of depression [15,24,25] and can also significantly predict the severity of subsequent depression [21].

Dysfunctional attitudes are ‘unspoken abstract norms, abstract standards by which an individual judges their own worth’ and by which they can judge others [30]. As in the case of younger adults, the use of a catastrophic coping style [31,32] and a focus on negative information have been associated with depressive symptoms in older adults [33]. It has been shown that increasing age leads to more dysfunctional attitudes [34], and this association is also present in people aged 60 and over [35,36]. Since dysfunctional attitudes are prevalent in this population and are associated with depression [36], an assessment of dysfunctional attitudes in this population can give us indications of the risk of depression.

The prevalence of dysfunctional attitudes among older adults is very significant, with several studies reporting that there is a strong association between dysfunctional attitudes and the onset of depression. Older adults often demonstrate maladaptive cognitive patterns, which can exacerbate feelings of hopelessness and contribute to the development of depressive symptoms [37]. These dysfunctional attitudes, characterised by negative self-perceptions and unrealistic expectations, are prevalent in this demographic group and can lead to increased vulnerability to depression [38], and so, addressing these dysfunctional attitudes is crucial for the effective treatment of depression in older adults [39].

Older adults with maladaptive beliefs are at greater risk of developing depression, emphasising the need to have an instrument that can assess these beliefs that can give rise to dysfunctional attitudes [38,40].

Weissman and Beck [31] and Beck [19] developed the Dysfunctional Attitudes Scale (DAS) to assess negative attitudes perceived as predisposing factors for psychopathology (mainly depression), and the measure has acceptable reliability and validity [41]. The DAS makes it possible to assess the cognitive processing characteristic of depression, identifying the underlying dysfunctional beliefs that constitute a vulnerability factor for depression [31]. The DAS was originally a 100-item scale, and Weissman [42] developed two shorter versions of 40 items, the DAS-A and DAS-B, which have been widely used in clinical practice.

To develop the reduced versions (DAS-A and DAS-B), the 100-item DAS was subjected to a factor analysis to identify which reduced version might be more feasible in terms of the time needed to complete the measure without a loss of reliability. Ten factors were extracted from the 100 items. In the revision process, Weissman assumed a unidimensional approach to dysfunctional beliefs, deriving the first extracted factor that identified the most general dimension and contributed with the maximum common variance among all items. The results of the factor analysis were used to construct the DAS-A and DAS-B [42].

Gouveia [41] validated the Portuguese version of the 40-item DAS-A and studied its reliability and validity, obtaining acceptable test–retest reliability (r = 0.69) and good internal consistency (Cronbach’s alpha = 0.84). Rojas et al. [43,44] developed the DAS-18A and DAS-18B versions for Germany, which showed good internal consistency (Cronbach’s alpha DAS-18A = 0.80; Cronbach’s alpha DAS-18B = 0.89) for total values. In our study, we opted for the DAS-18B for two reasons, namely the slightly better Cronbach’s alpha and the composition of the questions for use in the older population. The DAS 18-A includes questions that older adults perceive as not relevant to their current lives (e.g., “If I fail at work, then I am a failure as a person.”; “If I ask a question, it makes me look inferior.”). The 18 items of the German DAS-18B were translated and adapted for the Portuguese population in Gouveia’s study [41].

Some authors report that, in addition to the total score of the questionnaire being considered a predictor of depression, it is important to consider the tendency to give extreme answers in Likert-type questionnaires. The extreme answers are, for example, one or seven on a Likert scale of one to seven [45,46]. It is important to distinguish between extreme negative and positive responses, where an extreme negative response shows complete agreement with dysfunctional items and total disagreement with functional items in the questionnaire. In turn, extremely positive responses represent complete agreement with functional items and complete disagreement with dysfunctional items in the questionnaire [46,47,48]. For example, total agreement with the statement ‘It’s possible to earn someone else’s respect without being especially talented at anything’ is an example of an extremely positive response, as is total disagreement with the statement ‘If I fail at my job, then I’m a failure as a person’ [48]. Some studies report that the way individuals respond (extremely negative or positive answers) to the DAS questionnaire can give clues as to who will develop depression or relapse [45,46,49]. However, this association is not clear, as possible cultural and language differences may influence the extreme response [48].

The use of the DAS can be an important aid in the diagnosis and prognosis of depression, as patients bring with them complex and unique sets of beliefs, attitudes, prejudices, assumptions, and cognitions that influence their problems and the chances of a more favourable outcome [48,49,50,51]. We found no other instruments validated for Portugal that assess the same construct as the DAS-18B.

We chose to carry out this study with participants aged 60 or over, since depression is the most common psychiatric disorder in the elderly, which translates into a significant and growing public health problem, specifically in this population [52,53].

Depression in older adults generally has different symptoms compared to younger adults. Older adults may show more somatic symptoms, such as fatigue, sleep disturbances, functional impairment, and reports of physical pain, rather than the emotional symptoms considered more classic, such as sadness or hopelessness, as happens at younger ages [54,55]. In addition, cognitive deficits, including memory loss and difficulty concentrating, are more pronounced in older adults, which can potentially make it more difficult to diagnose depression [56].

Older adults may be less likely to report feelings of sadness, instead expressing their distress through irritability or withdrawal from social activities [57]. This manifestation of more specific symptoms may also be associated with the stigma surrounding depression, starting with self-stigma, which exacerbates feelings of worthlessness and isolation, which can lead to a decrease in help-seeking behaviour, further worsening depressive symptoms in this demographic group [58].

The impact of depression on quality of life differs significantly between older and younger adults. In older adults, depression is associated with cognitive decline and decreased independence, severely affecting their ability to perform daily tasks and their overall quality of life, which translates into increased functional disability for these people [59,60]. Although both age groups show a negative correlation between depression and quality of life, the specific domains affected may vary. In other words, in older adults, the challenges they face are more often associated with the cognitive domain, and younger adults mainly experience impacts in the physical health domain [61,62].

This divergence in depressive symptomatology in older adults can lead to underdiagnosis or misdiagnosis in this population, as traditional assessment tools may not identify these more specific characteristics [63].

Its prevalence is estimated to be close to 15 per cent among older adults in the general population, and the global population of adults aged 60 and over is expected to double between 2015 and 2055 [64]. On the other hand, dysfunctional attitudes can be predictors of depression, both for initial episodes and relapses [65,66], which is why we chose to study the DAS 18-B scale in older adults, in order to have an instrument that assesses dysfunctional attitudes in this population, but which can also be useful as a predictor of depression. In addition, older adults tend to receive mental health care more often from the general medical sector (e.g., primary care health professionals) than from specialised providers (e.g., a psychiatrist or psychologist) [53], and that is why it is very important that new assessment tools are developed with this scenario in mind.

The aim of the study was to assess the psychometric properties of the Portuguese version of the 18-item Dysfunctional Attitudes Scale (Form B) in a Portuguese sample of people aged 60 or over.

## 2. Materials and Methods

### 2.1. Setting and Data Collection

The data for this cross-sectional study was collected from native speakers of Portuguese in Portugal during a face-to-face assessment in a community setting between April and June 2023. We used the STROBE Checklist for cross-sectional studies [67]. This study was approved by the Ethics Committee of the University of Évora (Reference no. 22073) and complies with all the ethical principles recommended in the Declaration of Helsinki.

### 2.2. Patient and Public Involvement

Participants were involved in (targeted) data collection. The representatives of the stakeholders and public groups were not involved in any other stage of the study. The main conclusions of the study will be made publicly available online in Portuguese and English and in open access, as it has the advantage of allowing the results to be made available to everyone.

### 2.3. Sample

Convenience sampling was used. The sample size was 207 participants, an adequate size for this type of study, calculated on the basis of a minimum of 10 participants for each item in the measurement instrument [68,69,70,71]. The ages of the participants in this study ranged from 60 to 96, with an average age of 68.79 years (SD = 6.514), the majority being female (58.5%), the majority retired (60.9%), the majority married/married in law (76.8%), the majority having attended school but not higher education (80.6%), and the majority living with their spouse/partner (77.4%). All these sociodemographic characteristics can be seen in Table 1 (Descriptive characteristics of the study sample). The inclusion criteria were that participants were natives of Portugal, Portuguese speakers, able to read and write, functionally autonomous, and living in the community in their usual residences.

### 2.4. Measure

#### 2.4.1. Sociodemographic Questionnaire

The socio-demographic questionnaire included information on the study participants, such as gender, age (years), professional status, marital status, schooling, and ‘who they live with’.

#### 2.4.2. Dysfunctional Attitudes Scale-18B (DAS-18B)—Portuguese Version

The Dysfunctional Attitudes Scale is a self-report questionnaire designed to assess and identify dysfunctional attitudes, thoughts, and schemas associated with depression [43,44]. The original version of the DAS-18B [43,44] consists of 18 items answered on a seven-point Likert scale (1 = strongly agree to 7 = strongly disagree). A higher total score indicates a greater presence of dysfunctional attitudes. The questionnaire is constructed so that higher scores indicate more dysfunctional attitudes of respondents. Total scores can range from 18 to 126 [43,44]. The internal consistency of the original study was good (Cronbach’s alpha = 0.89) with a two-factor structure of (1) attitudes towards “performance evaluation” (n = 8, items 1, 3, 4, 5, 6, 9, 10, and 11) and (2) attitudes towards “recognition by others” (n = 3, items 15, 16, and 18). Seven items were not assigned to any factor (2, 7, 8, 12, 13, 14, and 17) and are not included in the subscales, and three items (2, 14, and 17) were revealed [43,44]. The questionnaire is constructed so that the higher the overall score, the more dysfunctional the respondent’s attitude is. The total score can range from 18 to 126 [43,44]. In the present study, we used a version of the DAS-18B, whose 18 items had already been translated into Portuguese in the validation study of the 40-item DAS for the Portuguese population by Gouveia [41]. To support the comparability of the results, we chose to maintain the overall composition and item wording of the original DAS-18B [43,44], with the translation and adaptation performed by Gouveia [41] for the Portuguese population.

#### 2.4.3. Beck’s Depression Inventory (BDI-II)

This instrument was developed by Beck, Ward, Mendelson, Mock, and Erbaugh [11], and is a self-report questionnaire. This scale was adapted for the Portuguese population by Martins, Coelho, Ramos, and Barros [72], and its psychometric properties have been improved since its validation. The psychometric qualities of the BDI-II show adequate levels of internal consistency, considered by some authors as “excellent” (Cronbach’s alpha = 0.91), and high test–retest reliability (r = 0.93; *p* < 0.001) [72]. This instrument aims at distinguishing depressed and non-depressed individuals, as well as measuring the severity of depressive symptoms. It is made up of 21 items grouped into three factors, namely the 1—cognitive factor (n = 8); 2—affective factor (n = 6); and 3—somatic factor (n = 7). There are four response options for each item (except for items 16 and 18, for which there are seven response options). The severity of symptoms over the past two weeks is rated on a four-point Likert scale, (e.g., sadness, 0—I do not feel sad; 3— I’m so sad or unhappy that I can’t stand it anymore) [73]. Scores less than or equal to 13 indicate minimal symptomatology; between 14 and 19 mild depression; 20 to 28 moderate depression; and 29 to 63 severe depression [74].

#### 2.4.4. Analysis

In our study, the items were not translated and culturally adapted because a Portuguese version of the DAS already existed [41], from which the 18 items included in the DAS-18B were extracted. The data were analysed and processed using the Statistical Package for Social Sciences software (SPSS^®^) version 28.0.0.0 (190) for Windows.

## 3. Results

### 3.1. Study Sample and Descriptive Statistics

A total of 207 people, living in the community in their usual residences and functionally autonomous, participated in the study. The descriptive characteristics of the sample are shown in Table 1.

To verify the validity of the application of factor analysis, the KMO (Kaiser–Meyer–Olkin Measure of Sampling Adequacy) and Bartlett’s test of sphericity were assessed. The KMO value obtained was average at 0.723. Bartlett’s test presented a value of χ^2^ (55) = 641.07, with statistical significance (*p* < 0.001), indicating that the variables presented significant inter-correlations (Table 2). Therefore, based on the information mentioned above, it can be stated that the scale is suitable for the development of a factor analysis with the current sample.

An exploratory factor analysis was performed with the 18 items of the scale, and six factors were obtained with an eigenvalue greater than one, which explained 64.61% of the total variance. However, the factor structure obtained proved to be very different from the author of the scale [43,44], and internal consistency values of the last three factors of less than 0.6 were not acceptable for the continuation of the study.

Thus, it was decided to conduct the analysis in accordance with [43,44], excluding items 2, 7, 8, 12, 13, 14, and 17. Subsequently, an exploratory factor analysis was performed on the DAS-18B scale (Table 2), in which three factors were obtained with an eigenvalue greater than one (Kaiser’s criterion). However, this analysis did not reproduce the results obtained by Rojas et al. [43,44], as the scale items were grouped, at the factor level, differently, having obtained three factors instead of two, as in the same author’s study. The three factors identified, with eigenvalues greater than one, jointly explained 58.95% of the total variance. The first factor represented 31.27% of the total variance of the scale, with an eigenvalue of 3.44 and composed of five items. The second factor contributed 15.67% of the total variance, presenting an eigenvalue of 1.72 and containing three items. The third factor, composed of three items, explained 11.99% of the total variance and had an eigenvalue of 1.32 (Table 3).

During the exploratory factor analysis, we chose to use the principal components method with varimax orthogonal rotation. In this process, three factors were obtained (Table 4). For this identification, only items with a factor loading greater than 0.30 were considered, according to [75].

Factor I was named “Performance evaluation based on results”. Factor II was named “Need for recognition by others”, and factor III was named “Performance evaluation based on demand for oneself” based on the reference studies [8,9,10,11,12,41,43,44]. To decide on the naming of the factors, there was a comparison between the empirical data and the factors proposed by Rojas [43,44].

With regard to factor I “Performance evaluation based on results”, in fact, establishing rigid and unrealistic rules to guide and evaluate one’s life predisposes the person to depression, since dysfunctional attitudes predispose the person to high levels of stress with a greater dependence on results [76,77]. Perfectionism as a self-imposed rule for performance perceived as satisfactory by the individual is associated with an increase in depressive feelings when this perfectionism is not achieved [77,78], as well as other psychiatric conditions [77,79,80]. Excessive self-belief in perfection and a low tolerance for one’s own failings can be associated with depressive symptoms, to the extent that depression is often associated with high levels of desire for perfectionism in performance [81,82]. The relationship between perfectionist performance concerns and depressive symptoms can be reciprocal, i.e., concerns about perfectionist performance are associated with an increase in depressive symptoms and vice versa [83]. Health professionals must take into account this reciprocal relationship between perfectionist performance concerns and depressive symptoms so that valuable information is not lost in the assessment and treatment of patients [83]. A recent systematic review points in the same direction, i.e., inadequate perfectionism in relation to performance is consistently associated with depression [84].

With regard to factor II “Need for recognition by others”, the literature points out that the fear of not being recognised by others can be associated with depressive symptoms [81,82]. If the individual’s perception is that important people have a negative view of them, this can be associated with depressive symptoms. Conversely, if the individual’s perception is that important people have a positive view of them, then this can be a protective factor against depression [85]. The excessive search for recognition from others can contribute to the appearance of depressive symptoms [86], so that even the facial expressions of others are taken into account by the individual [87]. Past experiences with others are also important, as memories of shame with other people can trigger an avoidance of contact with others [88].

With regard to factor III “Performance evaluation based on demand for oneself”, if the individual has inflexible beliefs and thoughts of self-demand and if there is no flexibilization of these thoughts and beliefs, this can lead to the appearance of depressive symptoms [89]. Depressed patients tend to describe fewer positive aspects of themselves and focus more on negative aspects. In fact, negative self-perceptions are a characteristic of depression [85], and in addition, people who are depressed can devalue their relative importance to other people [90]. The thought of preventing others from knowing how the individual “really is” because it is necessary to “hide” can also be associated with depressive symptoms [88,91], especially if there are memories of shame involving the other [91,92]. A high level of importance given to interpersonal relationships and the other person’s perspective, as well as the insecurity the individual feels in relation to the other, can be associated with depressive symptoms [93,94,95]. Comparison with others and the perceived importance of the interest that others may have in oneself can be associated with depressive symptoms [95,96].

### 3.2. Construct Validity

The correlations between the factors of the DAS-18B scale proved to be positive and statistically significant (Table 5). These results indicate that the items belonging to the same factor have content homogeneity, which confirms the construct validity.

Regarding the factor “Performance evaluation based on results” and the factor “Performance evaluation based on demand for oneself” there was a positive and moderate correlation coefficient *(r* = 0.449). The factor “Performance evaluation based on results” was also significantly correlated with the factor “Need for recognition by others”, with Pearson’s correlation coefficient being positive and low (*r* = 0.203). Finally, the coefficient of association between the factors “Need for recognition by others” and “ Performance evaluation based on demand for oneself” was also positive and very weak (*r* = 0.166).

### 3.3. Reliability

The reliability of the 11 items of the DAS-18B Scale was reasonable, with a Cronbach’s alpha of 0.770 and a retest value of 0.717. Regarding the retest, Cronbach’s Alpha values ranged from 0.656 to 0.745. Using McDonald’s omega coefficient, a value of 0.755 was obtained, and for the retest, it was 0.681. Cronbach’s alpha and McDonald’s omega coefficient were used to test the reliability of the DAS-18B scores. Regarding the factors obtained, the alpha value for the factor “Performance evaluation based on results” was reasonable (0.766), “Need for recognition by others” (0.681) and “Performance evaluation based on demands on oneself” (0.651) were weak but acceptable. As McDonald’s omega is calculated on the basis of non-standardised item loadings, it gives higher and more realistic results than Cronbach’s alpha [97]. The McDonald’s omega for factor 1 was 0.769, for factor 2 0.694, and for the last factor 0.703. The Cronbach’s alpha values and the McDonald’s omega coefficient indicated an adequate internal consistency for the study.

We chose to keep all items because the removal of items would not change, considerably, the scale’s alpha (Table 6). That is, if item 15 was to be removed in the test and item 9 in the retest, there would be little change in the alpha coefficient (Table 6).

Regarding the factors obtained, the alpha value referring to the factor “Performance evaluation based on results” was reasonable (0.766). The “Need for recognition by others” (0.681) and “Performance evaluation based on demand for oneself” (0.651) were weak but acceptable, which is likely attributable to the low number of items comprising each subscale. Thus, it may be said that Cronbach’s alpha values indicated an internal consistency that is adequate for the pursuit of the study.

### 3.4. Convergent Validity

To evaluate the convergent validity of the DAS-18B scale, Pearson’s correlation coefficients were calculated between the three factors of the DAS-18B and the three factors of the BDI-II. Table 7 indicates the values of the correlation coefficients obtained, as well as their levels of significance.

Regarding the first factor of the DAS-18B scale, called “Performance evaluation based on results”, it was significantly correlated with the somatic dimension of the BDI-II (*r* = −0.191), with the correlation coefficient being negative and very low (Table 7). The second factor “Need for recognition by others” of the DAS-18B scale showed a negative and statistically significant correlation with all factors of the BDI-II scale. The correlation coefficient was very low in relation to the cognitive dimension (*r* = −0.155) and low in relation to the affective dimension (*r* = −0.283) and somatic dimension (*r* = −0.260) (Table 7). Finally, the factor “Performance evaluation based on demand for oneself” also revealed a statistically significant negative correlation with all dimensions of the BDI-II scale, with a low correlation coefficient with the cognitive dimensions (*r* = −0.338) and affective (*r* = −0.338), and moderate considering the somatic dimension (*r* = −0.424) (Table 7).

### 3.5. Intra-Rater Reliability

An intra-rater reliability study was carried out by applying the same assessment instrument on two different occasions to the same patient ((ICC = 0.989); F(69.69) = 188.011; *p* < 0.001)). When analysed, there was very good agreement, demonstrating that it is a reliable instrument (Table 8).

## 4. Discussion

The study sought to evaluate the psychometric properties of the Portuguese version of the 18-item Dysfunctional Attitudes Scale (Form B), analysing its construct validity, reliability, and alternative factor structure, as well as its convergent validity with the BDI-II. Our study shows that this instrument has adequate psychometric properties and that the translation into Portuguese is reliable and valid. Three factors were obtained, explaining 58.98 per cent of the total variance, which according to Marôco [98], fulfils this criterion because it has a value of more than 50%. As with the original development of the DAS18-B [43,44], seven items were not integrated into any factor. No other validation studies of this instrument were found in other countries, apart from the study by Rojas et al. [43,44].

The content validity obtained positive and statistically significant results, which indicates content validity. The reliability analysis was reasonable, with a Cronbach’s alpha of 0.770 for the test and 0.717 for the retest. The alpha values of the factors “Need for recognition by others” (0.681) and “Performance evaluation based on demand for oneself “ (0.651) were shown to be weak but acceptable according to the criteria of Hill and Hill [99]. With regard to convergent validity, the DAS-18B demonstrated significant correlations with the BDI-II, particularly for the subscale “Performance evaluation based on demand for oneself“, which is in line with the [43] study, demonstrating that the BDI-II is a suitable instrument to assess convergent validity [43,44,100].

The DAS-18B should be interpreted in terms of its dimensions, but also in terms of the total score of the sum of all the items, i.e., a higher total score of all the items indicates a greater presence of dysfunctional attitudes [43,44], which can be predictive of depression, both in the initial episode and in relapses [21,48,101]. We can conclude that the Portuguese version of the DAS-18B is a reliable and valid instrument, just like the original scale [43,44].

We searched for but did not find any studies evaluating the psychometric properties of the 18-item Dysfunctional Attitudes Scale (Form B) in other countries so that we could compare the results. DAS-18B is a predictor of depression [21,48,101], and we also know that many patients may have a self-stigma about depression [65,102,103]. In fact, self-stigma is associated with poor clinical and functional outcomes in severe mental illness [66], so the issue of self-stigma cannot be ignored.

In order to circumvent the possibility of patients’ self-stigma in relation to depression by applying instruments that directly assess depression, the use of the DAS-18B is useful for health professionals, and according to some authors, dysfunctional attitudes can be predictive of depression [15,21,24,25]. Thus, professionals can be alert to the possible development of depression in patients and take preventive measures in community settings and in follow-up consultations in primary health care and hospitals. However, our study does not allow us to definitively conclude the same as these authors.

A recent systematic review with a meta-analysis reported that dysfunctional attitude scores were notably high in people with bipolar disorder compared to healthy patients, suggesting a strong association between dysfunctional attitudes and mood states, namely depression [40].

On the other hand, a study involving older adults in Romania found no direct correlation between dysfunctional attitudes and depression, suggesting that the association may be influenced by other factors, such as stress [104].

With an initial assessment using the DAS-18B, it is possible to realise the possible development of depression and implement preventative measures, as we know that preventative measures in relation to depression and depressive symptoms are effective [105,106]. Furthermore, the existence of an 18-item version of the DAS for the Portuguese population is an indisputable advantage, since the reduced number of items reduces the distortion of results due to subject fatigue and can increase the willingness to give valid answers [43].

### Limitations

In the study sample, participants were aged 60 or over, so validation results at younger ages are uncertain. The instrument was only studied in a community setting, so results outside the same context are uncertain. We found no studies evaluating the psychometric properties of the 18-item Dysfunctional Attitudes Scale (Form B) in other countries so that we could compare the results, which limited the comparative analysis and subsequent discussion.

## 5. Conclusions

The Portuguese version of the DAS-18B showed adequate psychometric properties, with the advantage of being a short and easy-to-apply instrument. In addition, it can be a predictor of depression both in an initial episode and in relapses, although there is no complete consensus in the scientific community as to whether dysfunctional attitudes are predictive of depression.

This aspect is very useful for nurses’ clinical practice and for the overall management of healthcare, since Portuguese healthcare professionals have an instrument that assesses dysfunctional attitudes and, at the same time, can be, according to some authors, a predictor of depression, which makes its application in the context of health promotion in general and mental health promotion important. This instrument can be used as a predictor of depressive symptoms or relapses of depression, which is very important when developing work plans in the clinical practice of general care nurses and psychiatric specialist nurses, particularly in cognitive–behavioural approaches and, more recently, in the context of metacognitive therapy. Recognising dysfunctional attitudes can help patients reflect on their own thinking in the context of metacognitive therapy, where the aim is for patients to identify cognitive biases to promote more adaptive responses to internal experiences, thus minimising depressive symptoms.

In addition, older adults tend to receive mental health care more often in the general medical sector (e.g., primary care health professionals) than from specialised providers. So, it is very important that new assessment instruments are developed that take this reality into account, i.e., it is an instrument that can be used by nurses both in psychiatric services and in primary health care. It is recommended that in future research the instrument be tested on participants aged 18 and over to validate the study in other countries and other contexts, such as clinical settings.

## Figures and Tables

**Table 1:** Descriptive characteristics of the study sample.

Characteristics	N	%
Total sample	207	100
Gender		
Female	121	58.5
Male	86	41.5
Age (years)		
60–70	141	68.1
71–80	55	26.6
>80	11	5.3
Employment situation		
Employee	63	30.4
Unemployed	13	6.3
Retired	126	60.9
Retired on disability	4	1.9
Medical Leave	1	0.5
Marital status		
Single	10	4.8
Married/cohabiting	159	76.8
Widower	26	12.6
Divorced	12	5.8
School level		
Can read and write, but did not attended school	14	6.8
Attended school but not Higher education	167	80.6
Higher education	26	12.6
Who you live with		
Alone	28	13.5
Spouse/Partner	160	77.4
Son/Daughter	15	7.2
Brother/Sister	1	0.5
Other family member	3	1.4

**Table 2:** KMO and Bartlett’s test for DAS-18B.

Kaiser–Meyer–Olkin measure of sampling adequacy		0.723
Bartlett’s test of sphericity	Approx. chi-square	641.074
	χ^2^	55
	*p*	0.000

**Table 3:** Component matrix run from DAS-18B.

	Own Value	% of Variance	% Cumulative Variance	Own Value	% of Variance	% Cumulative Variance
1	3.440	31.271	31.271	2.732	24.840	24.840
2	1.723	15.665	46.936	1.916	17.422	42.262
3	1.319	11.987	58.923	1.833	16.661	58.923
4	0.941	8.556	67.479			
5	0.841	7.644	75.123			
6	0.740	6.728	81.850			
7	0.533	4.841	86.691			
8	0.466	4.232	90.924			
9	0.354	3.218	94.141			
10	0.343	3.121	97.262			
11	0.301	2.738	100.000			

**Table 4:** Principal component analysis of DAS-18B.

Items		Factors		
	Factor Assignment According to [43]	1	2	3
DAS-18B-Q4 (It is only worth doing what we are sure of doing well)	Achievement	0.839		
DAS-18B-Q5 (If I fail partially that is as bad as if I fail totally)	Achievement	0.737		
DAS-18B-Q11 (People with good ideas are worth more than others)	Achievement	0.703		
DAS-18B-Q3 (Asking for help is a sign of weakness)	Achievement	0.695		
DAS-18B-Q1 (It is foolish to take even a small risk, because if things go wrong the consequences can be disastrous)	Achievement	0.488		
DAS-18B-Q16 (My happiness depends more on others than on me)	Recognition		0.819	
DAS-18B-Q18 (It is very important what others think about me)	Recognition		0.769	
DAS-18B-Q15 (If others do not like us, we cannot feel happy)	Recognition		0.729	
DAS-18B-Q6 (If others know how you really are, they will have a worse impression of you)	Achievement			0.752
DAS-18B-Q9 (If I am not demanding of myself, I will probably end up as a mediocre person)	Achievement			0.731
DAS-18B-Q10 (To be a valid person, I have to be “really good” at something)	Achievement	0.436		0.682

**Table 5:** Pearson’s correlation between the scores of the DAS-18B factors.

		Need for Recognition by Others	Performance Evaluation Based on Demand for Oneself
Test	Performance evaluation based on results	0.203 **	0.449 **
	Need for recognition by others		0.166 *

Note: * *p* ≤ 0.05. ** *p* ≤ 0.01.

**Table 6:** Internal consistency—study the items of the DAS-18B.

	Total Item Correlation Corrected	Total Item Correlation Corrected Retest	Cronbach’s Alpha If the Item Is Deleted	Cronbach’s Alpha If the Item Is Deleted Retest
DAS-18B-Q4	0.446	0.598	0.750	0.656
DAS-18B-Q5	0.608	0.529	0.729	0.672
DAS-18B-Q11	0.605	0.579	0.726	0.659
DAS-18B-Q3	0.453	0.282	0.749	0.708
DAS-18B-Q1	0.357	0.266	0.760	0.711
DAS-18B-Q16	0.362	0.345	0.759	0.700
DAS-18B-Q18	0.307	0.278	0.766	0.710
DAS-18B-Q15	0.207	0.366	0.775	0.697
DAS-18B-Q6	0.303	0.277	0.765	0.709
DAS-18B-Q9	0.384	0.024	0.758	0.745
DAS-18B-Q10	0.558	0.440	0.735	0.685

**Table 7:** Correlations between the DAS-18B and BDI II Factors.

	Cognitive Dimension	Affective Dimension	Somatic Dimension
Performance evaluation based on results	−0.071	−0.120	−0.191 **
Need for recognition by others	−0.155 *	−0.283 **	−0.260 **
Performance evaluation based on demand for oneself	−0.338 **	−0.338 **	−0.424 **

Note: * *p* ≤ 0.05. ** *p* ≤ 0.01.

**Table 8:** Intra-rater reliability.

	Intraclass Correlation	Confidence Interval 95%		F-Test with True Value 0			
		Lower Limit	Upper Limit	Value	df1	df2	Sig
Unique measurements	0.987	0.980	0.992	157.416	69	69	0.000
Average measurements	0.994	0.990	0.996	157.416	69	69	0.000

## Data Availability

The data presented in this study are available upon request from the corresponding author.

## Public Involvement Statement

No public involvement in any aspect of this research.

## Guidelines and Standards Statement

This manuscript was drafted against the Strengthening the Reporting of Observational Studies in Epidemiology (STROBE) for cross-sectional studies.
